# Poly(vinyl methyl ether) hydrogels at temperatures below the freezing point of water—molecular interactions and states of water

**DOI:** 10.1007/s00396-014-3283-z

**Published:** 2014-06-18

**Authors:** Marcin Pastorczak, Gustavo Dominguez-Espinosa, Lidia Okrasa, Marek Pyda, Marcin Kozanecki, Slawomir Kadlubowski, Janusz M. Rosiak, Jacek Ulanski

**Affiliations:** 1 Department of Molecular Physics, Lodz University of Technology, Zeromskiego 116, 90-924 Lodz, Poland; 2 Technological Lab of Uruguay (LATU), 6201 Av. Italia, 11500 Montevideo, Uruguay; 3 Department of Chemistry, Rzeszow University of Technology, Powstancow Warszawy 6, 35-959 Rzeszow, Poland; 4 Institute of Applied Radiation Chemistry, Lodz University of Technology, Wroblewskiego 15, 93-590 Lodz, Poland; 5 Present Address: Institute of Experimental Physics, University of Warsaw, Hoza 69, 00-681 Warsaw, Poland

**Keywords:** PVME hydrogels, Raman spectroscopy, Dielectric spectroscopy, DSC, Molecular interactions, Water pre-melting

## Abstract

Water interacting with a polymer reveals a number of properties very different to bulk water. These interactions lead to the redistribution of hydrogen bonds in water. It results in modification of thermodynamic properties of water and the molecular dynamics of water. That kind of water is particularly well observable at temperatures below the freezing point of water, when the bulk water crystallizes. In this work, we determine the amount of water bound to the polymer and of the so-called pre-melting water in poly(vinyl methyl ether) hydrogels with the use of Raman spectroscopy, dielectric spectroscopy, and calorimetry. This analysis allows us to compare various physical properties of the bulk and the pre-melting water. We also postulate the molecular mechanism responsible for the pre-melting of part of water in poly(vinyl methyl ether) hydrogels. We suggest that above −60 °C, the first segmental motions of the polymer chain are activated, which trigger the process of the pre-melting.

## Introduction

Various states of water, whose physical and chemical properties are modified by interactions with a solute, have been discussed and studied since the beginning of twentieth century [1]. It is claimed that the states of water interacting with a solute differ from those existing in bulk water in their “structure”, rotational, and vibrational dynamics and freezing temperature. One of the first observations of this distinct water in water-polymer mixtures was performed with calorimetric method [2]. It has been noticed that the enthalpy of melting of ice in water-polymer mixtures is significantly lower than that, which should result from the analytical content of water. It was hence concluded that the interactions with a polymer prevent some fraction of water from freezing. The presence of liquid water below 0 °C in polymer systems has been confirmed by X-ray experiments [3], infrared spectroscopy [4], and, more recently, by broadband dielectric spectroscopy (BDS) [5, 6]. The detection in the last method bases on the presence of dielectric depolarization (at around 18 GHz), which results from rotational reorientation of non-freezing water molecules. The non-freezing water is often linked with the water, which has rotational or translational motions restricted at room temperature due to its interactions with a solute. Although the presence of non-freezing water in polymeric solution has been confirmed with various experimental techniques, the exact relations (both qualitative and quantitative) between the non-crystallized water, the rotationally restricted water, and the water hydrogen bonded to solute molecules are still not well understood.

In this work, we analyze an evolution of intermolecular interactions between water and selected model polymer at temperatures below the freezing point of water with use of Raman spectroscopy. Then, we relate the observed evolution of these interactions with changing fraction of non-crystallized water determined by means of dielectric spectroscopy and differential scanning calorimetry.

As a model system for these studies, we have chosen series of hydrogels with various crosslinking degree prepared by electron-beam irradiation of poly(vinyl methyl ether) (PVME) [7]. PVME is an amphiphilic amorphous polymer, and its aqueous solutions reveal a number of interesting properties, particularly the presence of the lower critical solution temperature (LCST) around 37 °C [8]. Our previous work was devoted to molecular relaxations at temperatures between −60 and 50 °C in the PVME hydrogels; in particular, we have studied the transition revealed around −18 °C, whose origin had been so far unclear [9]. We have shown therein that this transition is related to the pre-melting of water induced by the segmental motions of the polymer. Such phenomenon should be associated with modification of intermolecular interactions between polymer network and water in this temperature range; this hypothesis was one of motivations for the research described in the present work.

The selection of PVME hydrogels for these studies gives us an opportunity to investigate an influence of the crosslinking degree on the content of non-freezing and pre-melting water. Moreover, we would like to compare how these two types of water differ in their thermal properties, rotational motions, and ability to form hydrogen bonds with the polymer in the range of temperatures below the freezing point of water.

## Experimental

### Material description

Polymer hydrogels were prepared of 50 wt% aqueous solution of PVME (Sigma-Aldrich Co.). The crosslinking was performed by mean of electron-beam irradiation. Details of the radiation synthesis as well as of the sol-gel analysis may be found in our earlier papers [7, 10, 11]. The basic physical properties of studied samples are summarized in Table 1. All the samples were studied in their equilibrium swelling degree. The equilibrium swelling degree (ESD) is defined here as:

**Table 1:** Basic physical properties of studied samples

Sample name	Radiation dose [kGy]	Equilibrium swelling degree [g/g]	Polymer fraction *w*_PVME_
PVME-22	22	21.70	0.0440
PVME-35	35	9.02	0.0642
PVME-42	42.5	7.5	0.0998
PVME-50	50	6.36	0.1176
PVME-65	65	6	0.1358

1 $$ \mathrm{ESD}=\frac{m_{\mathrm{gel}}-{m}_{\mathrm{p}}}{m_{\mathrm{p}}} $$

where *m*_p_ is a weight of dry polymer network and *m*_gel_ is a weight of a swollen hydrogel at a time when its equilibrium swelling is attained. The polymer faction *w*_PVME_ we define as ratio of *m*_p_ to *m*_gel_.

### Experimental techniques

Recently, it has been reported that the kinetics of the crystallization of water and formation of various crystalline forms in PVME/water mixtures strongly depends on the applied cooling rate [3, 12]. It was therefore crucial to apply similar cooling procedures in all our experiments to be allowed to compare directly the results concerning the crystallized and the non-crystallized water. Furthermore, Zhang et al. [3] have reported that above −30 °C, the crystallization temperature of water in PVME/water mixtures strongly depends on the crystallization rate and is almost independent below this temperature. For this reason, in our experiments, the hydrogel samples have been annealed for at least 40 min at the temperature of −50 °C or lower.

Raman spectra were acquired with use of JobinYvon T64000 triple-gratings spectrometer equipped with the Olympus BX40 confocal microscope. The Ar-ion laser (LEXEL) line (514.5 nm) was used as a source of sample excitation. Laser power (4–6 mW at a sample) and time of a single measurement (240–360 s) were adjusted to obtain the high quality spectra. All Raman experiments were conducted with the same microscope objective (x50, N.A. = 0.5), and the laser light was focused 30–40 μm below the sample surface. Low temperature measurements (range −163 to +10 °C) were performed in helium-nitrogen cryostat, and liquid nitrogen was used as a cooling medium. Temperature was stabilized by Lakeshore 330 temperature controller with precision ±0.1 °C. Hydrogel samples were cooled down with a cooling rate over 10 °C/min. To minimalize the water loss (low pressure in the cryostat cell was needed for good temperature stabilization and could result in partial evaporation of water), the air was pumped out from the cell just after crystallization of water (detected by appearance of the ice band in the Raman spectrum). After reaching the temperature -163 °C, and after at least 40 minutes of isothermal annealing, the sample was measured during slow, step-like heating (each step was reached with 1–2 °C/min heating rate and then the sample was measured isothermally for 6 min). Due to such procedure, one can assume that the thermal histories of the samples measured by the Raman and by the dielectric spectroscopies were almost identical.

Differential scanning calorimetry (DSC) measurements were performed using TA2920 and Q1000 calorimeters (TA Instruments Inc.). The mass of inserted samples typically varied between 3 and 6 mg. Standard DSC measurements were performed for the temperature range from −50 to 55 °C. The cooling rate was 10 °C/min and heating rate was 3 °C/min.

Broadband dielectric spectroscopy (BDS) studies were performed using a Novocontrol® Alpha Analyser Concept 80 apparatus in the range of frequency 0.1–10^7^ Hz and in the temperature range −60 to +50 °C, with a temperature step of 3° (only the measurements conducted below 0 °C are the subject of this paper). Samples were placed between two gold-plated brass electrodes in the Novocontrol® Liquid Sample Cells BDS 1308. The liquid sample cell was used in order to maintain a good contact between the sample and the electrodes and to avoid water flowing out of a cell. The dimensions of the sample were 18-mm diameter and 1-mm thickness. A spacer ring 1 mm in thickness, 1-mm wide, and 20 mm in outer diameter, made of silica, was used to ensure stability of the dimensions of the samples.

In order to compare directly the integral intensities of the Raman bands the following mathematical procedure was applied:

- Each spectrum was normalized in a way that the total area under the curve equals one;
- The spectra were deconvoluted with PeakFit v4.12 (SeaSolve Software Inc., SYSTAT Software Inc.) assuming a presence of four components in the OH stretching region;
- The comparison of particular peaks related to different water states was performed on the basis on integral intensities estimated by the deconvolution.

## Results

### Raman spectroscopy studies

**Fig. 1:**
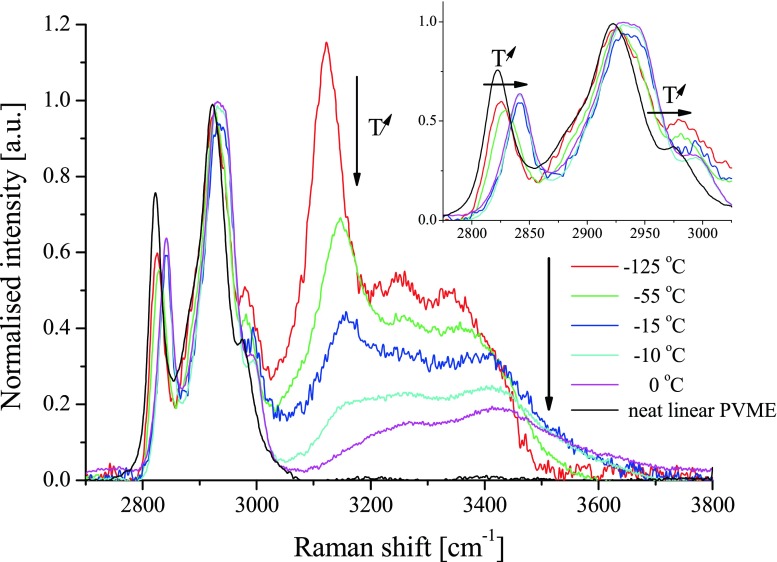
Raman spectra acquired on heating run at several temperatures −125, −55, −15, −10, and 0 °C for the sample PVME-65; spectra were normalized to the intensity of the polymer band at ca. 2,940 cm^**−**1^. The spectrum of neat linear PVME at room temperature is shown as a reference (*grey*). Changes in the intensities and positions of particular bands on heating are marked with *arrows*

Changes of the supramolecular structure of water and water interactions with PVME are well manifested in the spectral range 2,700–4,000 cm^−1^, as seen in the Raman spectra of the PVME-65 at several selected temperatures in Fig. 1. This range contains vibrational bands related to both stretching vibrations of CH_x_ of the polymer (bands located between 2,700 and 3,050 cm^−1^) and stretching vibrations of OH of water (in the range 3,100–3,700 cm^−1^) [11]. The intense narrow band at around 3,150 cm^−1^ is a well-known manifestation of a crystallized water, and it is referred to the “collective band of ice” (I_col_) [13]. A spectrum of a dry not-crosslinked PVME at 27 °C is additionally shown in the figure for a comparison. The shape of the multi-mode bands of CH_x_ stretching for the frozen hydrogel (2,700–3,050 cm^−1^, see the inset in Fig. 1) resembles well the corresponding bands for the dry PVME. It is particularly well seen for the symmetric CH_3_-stretching band (ν_s_(CH_3_)) at around 2,820 cm^−1^, which position is the same as the one in the totally frozen hydrogel at the temperatures between −125 and −55 °C. It is known from Maeda’s [14] and from our previous studies [11] that the position of this mode reflects the hydration of the PVME segments. In a neat, dry polymer, it is located at 2,820 cm^−1^, and in a fully hydrated polymer, it is at around 2,840 cm^−1^ [9, 14, 15]. The fact that this band shifts back to around 2,840 cm^−1^ after the cycle of cooling and heating implies also that no significant fraction of water was lost during the cycle, and all the samples studied returned to its initial hydration state.

Actually, the ν_as_(CH_3_) band (antisymmetric stretching, at around 2,970 cm^−1^ in a neat polymer) reveals similar hydration-dependence of the spectral position. Yet, the shift is not as clearly visible for the ν_as_(CH_3_) band as for the ν_s_(CH_3_) band because the former one is broader and less intense than the latter. These spectral features indicate that in the fully frozen hydrogel, below −55 °C, the polymer chain is dehydrated.

**Fig. 2:**
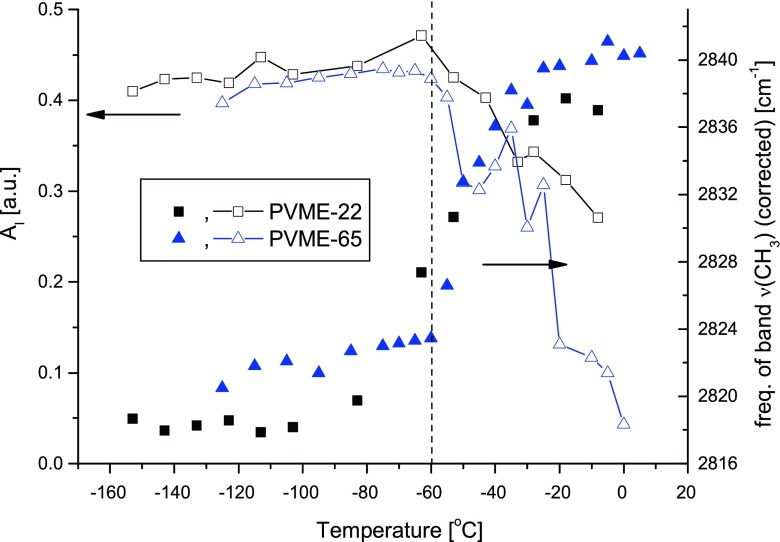
The temperature dependence of the position of the ν_s_(CH3) band of PVME (*full symbols*, right *y*-axis) and of the integral intensity of I_C_ band of ice (*empty symbols*, left *y*-axis) in the sub-zero range of temperature for sample PVME-22 (*squares*) and PVME-65 (*triangles*); *lines* connecting points are as guide for eyes; temperature of the change in the tendency is marked with the *dashed line*. One should note that according to Loozen et al. [15, 16] the ν_s_(CH_3_) band in the neat, uncrosslinked PVME shows a small temperature dependence, shifting to higher wavenumber by ca. 0.03 cm^**−**1^/deg with decreasing temperature. The data presented are therefore corrected for that temperature shift with respect to the wavenumber of the ν_s_(CH_3_) band at 25 °C

On heating of the sample, despite of the shift of the ν_s_(CH_3_) and ν_as_(CH_3_) bands to the higher wavenumbers also the center of the collective ice band at around 3,150 cm^−1^ shifts to the higher wavenumbers and the intensity of this band diminishes. The shift of the center of the collective band of ice with temperature is a well-known feature of the spectrum of the ice type I [13]. It is interpreted as the result of weakening of hydrogen bonds and coupling between adjacent OH oscillators. Hence, the similar effect observed in frozen hydrogels will not be discussed here. The evolutions of the integral intensity (A_I_) of the collective band of ice and of the position of the band ν_s_(CH_3_) on heating in the samples PVME-22 and PVME-65 are summarized in Fig. 2. It is apparent that in both samples, the values of integral intensity A_I_ are constant up to ca. −60 °C. Above this temperature, the value of A_I_ monotonously decreases down to its minimum at 0 °C when all the ice melts. This observation confirms an existence of the phenomenon of pre-melting of ice and points out that the pre-melting processes start already at around −60 °C.

One may also see in Fig. 2 that the position of the ν_s_(CH_3_) band and the value of A_I_ are both approximately constant at the lowest temperatures up to ca. −60 °C and above this threshold strong changes in both quantities occur. In both frozen hydrogels below ca. −60 °C, the ν_s_(CH_3_) is approximately equal 2,819 cm^−1^. That means that up to that temperature the PVME chains are dehydrated. Above −60 °C the ν_s_(CH_3_) band clearly shifts with temperature to higher wavenumbers. In the sample PVME-65 the center position of the ν_s_(CH_3_) band is around 2,840 cm^−1^ at ca. −20 °C indicating that the PVME chain is fully hydrated above this temperature. One can conclude that although at −20 °C, there is still significant fraction of ice (A_I_ = 0.1), the amount of pre-melted water in the sample is sufficient to reestablish the network of hydrogen bonds around polymer chains.

Taking into account the known dependence of the position of the ν_s_(CH_3_) band on the hydration of PVME segments at room temperature [6, 8, 16], we may estimate the local hydration degree of the polymer segments in the temperature range from −120 to 0 °C. We may assume that below 0 °C, the analyzed system is composed of two phases: the network of PVME chains surrounded by not frozen water molecules and the ice crystals. Then, the positions of ν_s_(CH_3_) should reflect the local concentration of PVME in the liquid phase ${w}_{{\mathrm{PVME}}_{\mathrm{local}}}$. When a fraction of PVME in a whole sample *w*_PVME_ is known (see Table 1), the fraction of liquid water in the whole sample *w*_H2O_^liq^ may be estimated using the following formula:

2 $$ {w}_{\mathrm{H}2\mathrm{O}}^{\mathrm{liq}}=\frac{w_{\mathrm{PVME}}\left(1-{w}_{{\mathrm{PVME}}_{\mathrm{local}}}\right)}{w_{{\mathrm{PVME}}_{\mathrm{local}}}} $$

**Fig. 3:**
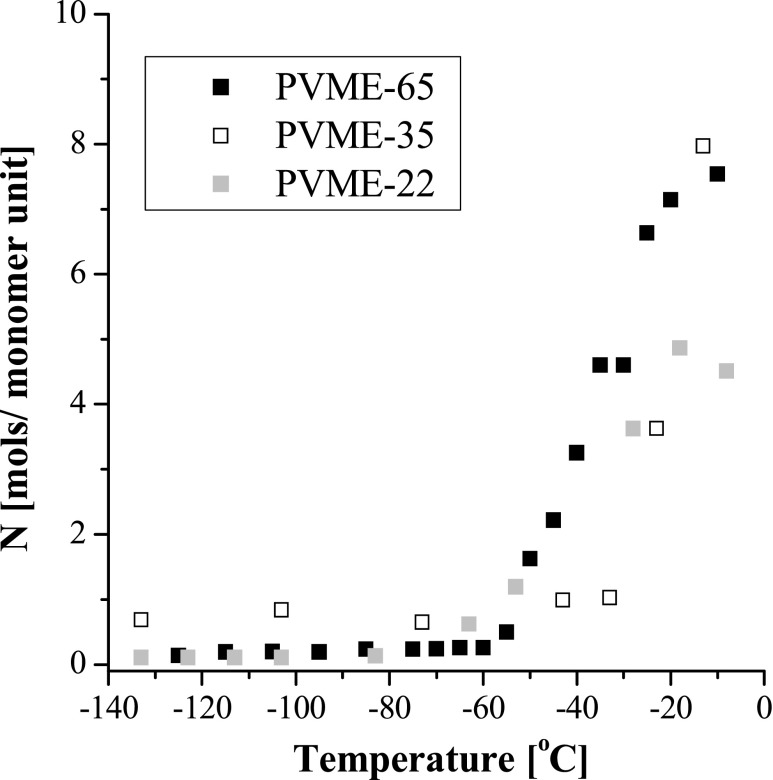
The hydration number *N* of the polymer monomer unit in samples: PVME-22 (*grey*), PVME-35 (*empty*), and PVME-65 (*black*) determined with use of Raman spectroscopy (see text)

The values of *w*_H2O_^liq^ determined in such a way were used to estimate the hydration number *N* (the number of water molecules in liquid state per monomer unit) in different temperatures with use of the formula (3):

3 $$ N=\frac{w_{\mathrm{H}2\mathrm{O}}^{\mathrm{liq}}}{w_{\mathrm{PVME}}}\cdot \frac{M_{\mathrm{PVME}}}{M_{\mathrm{H}2\mathrm{O}}} $$

where M_PVME_ = 58.08 is a molar mass of the monomer unit of PVME and M_H2O_ = 18.02 is a molar mass of water and *w*_PVME_ is a polymer fraction derived from sol-gel analysis (Table 1). We assume here that the chemical structure of the PVME monomer unit remains unchanged after radiation crosslinking. The results shown in Fig. 3 clearly indicate that for all samples studied, the hydration numbers are close to 0 below −60 °C, and above this temperature, they abruptly grow up to values 5–8 at −5 °C.

### Dielectric spectroscopy studies

**Fig. 4:**
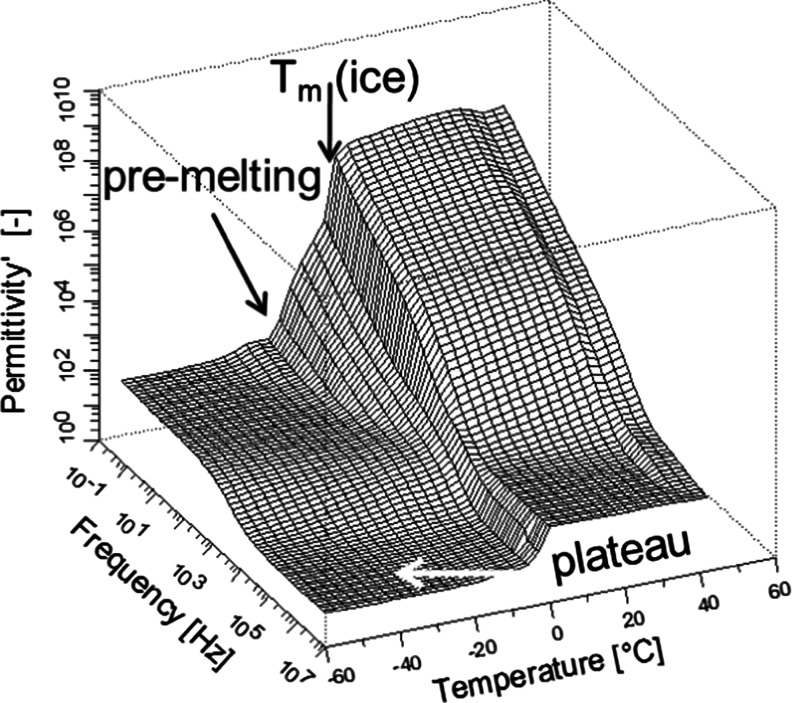
3D BDS plots of a real part of dielectric permittivity vs. frequency and temperature for hydrogel PVME-22; particular processes are marked by *arrows*

In our previous studies of molecular relaxations in PVME hydrogels with use of dielectric spectroscopy in the range 0.1–10^7^ Hz we have noticed an apparent *plateau* in the plot of the real part of the permittivity (*ε’*) at high frequencies (*f* > 10^5^ Hz) [5, 11] (Fig. 4). It is due to the reorientation of water dipoles at gigahertz and sub-gigahertz frequencies, which contributes to dielectric constant in the studied frequency range. Shinyashiki et al. [5] has studied polymer-water mixtures of different compositions (including aqueous solutions of linear PVME) with use of time domain reflectometry (TDR) in the temperature range −55 to +25 °C. They have shown that the dielectric process related to water reorientations decelerates from around 10^10^ Hz at −2 °C down to around 10^7^ Hz at −30 °C and has the major contribution to dielectric depolarization in the frequency range 10^5^–10^7^ Hz. In addition to the deceleration of liquid water band, they observed a drop in intensity of liquid water band at −5 °C, which was related to the crystallization of the major fraction of water. After partial crystallization of water, the remaining fraction of liquid water continued to decelerate its rotational reorientation on further cooling.

**Fig. 5:**
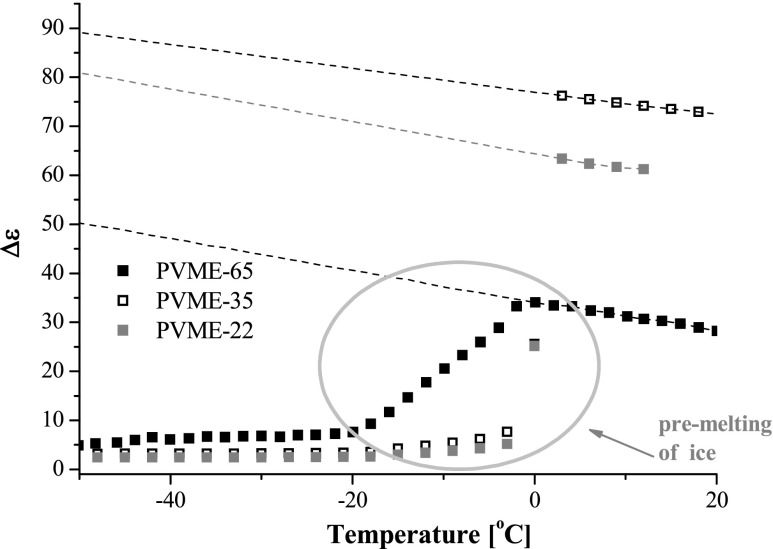
Dielectric strength at 1 MHz against temperature for PVME-22, PVME-35, and PVME-65; *dashed lines* denote linear extrapolation of ∆ε_all_

In the isochrones at 10^6^ Hz of PVME hydrogels, which are shown in Fig. 5, one can notice the peculiar changes in the values of dielectric strength *∆ε* with temperature; *∆ε* = *ε*_r_–*ε*_∞_, where *ε*_r_ and *ε*_∞_ are unrelaxed and relaxed values of dielectric constant, respectively. Given the fact that in the samples studied concentration of water is between 86 and 96 wt%., *∆ε* values were calculated assuming that *ε*_∞_ equals its theoretical value for liquid water, i.e., *ε*_∞_ = 1.78. For the samples PVME-22 and PVME-35, the dielectric strength slowly and monotonously increases from −20 up to −3 °C, and at −3 °C, the value of *∆ε* abruptly jumps. For the sample PVME-65, we may notice the monotonous growth of *∆ε* from −20 to 0 °C. From the Shinyashiki’s TDR studies [5], we may conclude that it is the non-crystallized water, which mostly contributes to the *∆ε* at 1 MHz. Hence, the value of the *∆ε* below −20 °C should correspond to the non-freezing water. The increase in the *∆ε* value between −20 and −3 °C we assign to pre-melting of water and abrupt jumps in *∆ε* at −3 °C to the melting of the remaining ice crystals. These results are in a very good agreement with our Raman studies, which demonstrates that at 0 °C all ice in PVME-65 is already melted (see Fig. 2). Both methods (BDS and Raman) show also that in PVME-22, significant fraction of ice starts to melt at 0 °C.

**Fig. 6:**
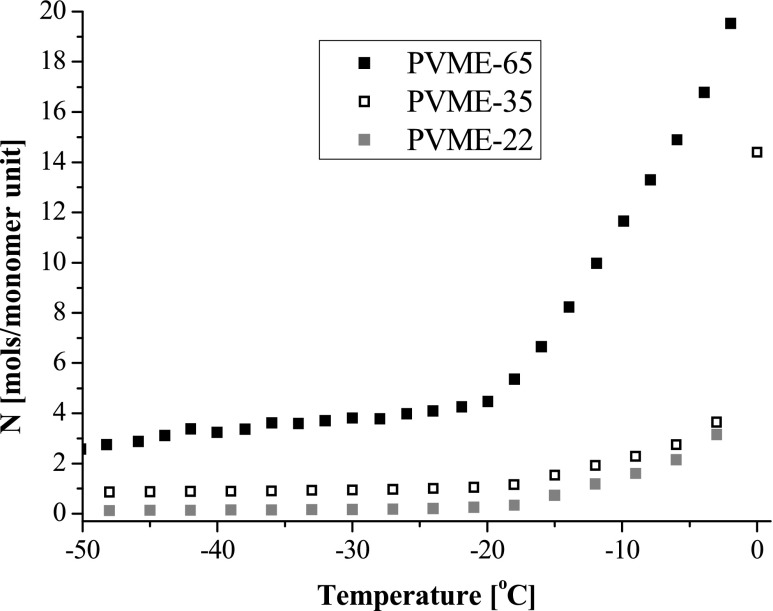
The hydration number *N* of the polymer monomer unit in samples PVME-22 (*grey*), PVME-35 (*empty*), and PVME-65 (*black*) determined with use of dielectric spectroscopy

Then, to relate the dielectric strength of water reorientation to the concentration of rotationally unrestricted water *c* in the sample, we have applied here the Cavell equation [17]:

4 $$ \varDelta \varepsilon =\left(\frac{\varepsilon }{2\varepsilon +1}\right)\left(\frac{N_Ac}{k_BT{\varepsilon}_0}\right)\cdot {\mu}_{\mathrm{eff}}^2 $$

where: *ε* is relative permittivity, *ε*_*0*_ the permittivity of vacuum, N_0_—Avogadro’s constant, k_B_—Boltzmann constant, and *μ*_eff_—an effective dipole moment. According to Eq. (2), the dielectric strength of the process is proportional to the number of rotating dipoles; in presented case *∆ε* at 1 MHz is proportional to *c.* The quantity of this water may be determined following the Shinyashiki’s assumption that *∆ε* would theoretically depend linearly on temperature also below the freezing point providing that all the water stays liquid. We may derive such fictitious value of *∆ε*_all_ by linear extrapolation of *∆ε* from the positive to the negative temperature range (the result of the extrapolation is marked in Fig. 5 with dashed lines). Taking the abovementioned assumptions, one can calculate a fraction of liquid water *w*_ucw_ in the hydrogels with the use of the following formula:

5 $$ {w}_{\mathrm{ucw}}=\left(1-{w}_{\mathrm{PVME}}\right)\cdot \frac{\varDelta s}{\varDelta {s}_{\mathrm{all}}} $$

where *w*_PVME_ is a weight fraction of polymer in a hydrogel determined with sol-gel method (see Table 1). The obtained liquid water fraction *w*_ucw_ can be easily recalculated into the hydration number *N* of polymer monomer unit with use of the formula (3) if we substitute *w*_H2O_^liq^ with *w*_ucw_. The BDS determined hydration number vs. temperature for PVME-22, PVME-35, and PVME-65 are plotted in Fig. 6. In all samples studied, the hydration number is practically temperature independent in the range from −50 to −20 °C and equals less than 1 for PVME-22 and PVME-35 and around 3 for PVME-65. *N* value monotonously grows between −20 and −3 °C and for PVME-22 and PVME-35 equals around 3 at −3 °C. Above −3 °C, the melting of ice takes place in these two samples. In the densely crosslinked PVME-65, we cannot detect typical fusion of ice; instead the fraction of liquid water steeply grows between −20 and 0 °C.

### Differential scanning calorimetry studies

**Fig. 7:**
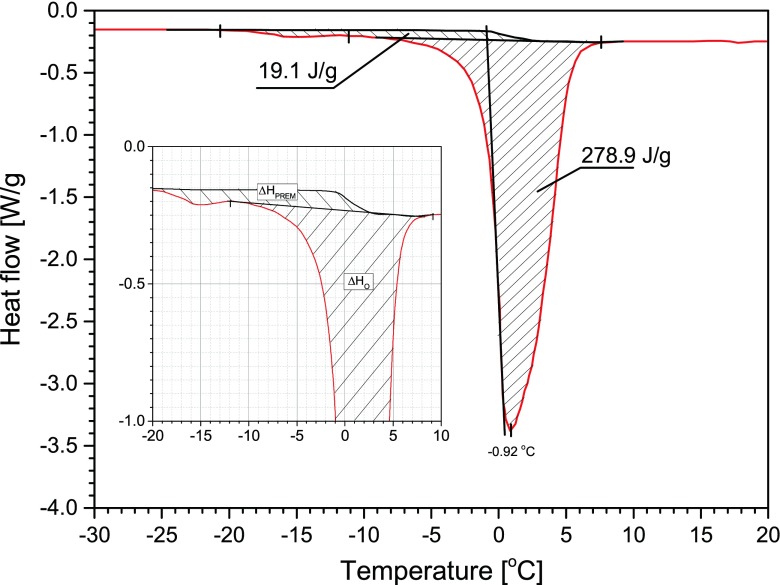
Exemplary graphical analysis of states of water in the PVME-22 hydrogel with marked bulk water (*upward diagonal fill*) and water which pre-melts on heating (*downward diagonal fill*); *inset*: focus on the pre-melting of water in DSC curve

DSC-based estimation of an amount of unfrozen water in a system presumes that the enthalpy of melting of ice *∆H*_all_ is proportional to amount of water which crystallizes. In the case of the PVME hydrogels, the values of *∆H*_all_ (normalized to the fraction of water in a sample) are always lower than the enthalpy of melting of ice for distilled water *∆H*_H2O_. This indicates the presence of some fraction of not frozen water below 0 °C in these systems. The exemplary thermogram (second heating run) for the PVME hydrogel is presented in Fig. 7. The detailed analysis of the thermogram allows distinguishing three different states of water present in a sample:

- “Normal” bulk water, which melts on heating around 0 °C, with enthalpy *∆H*_o_; in Fig. 7, it is marked by left-slanting lines.
- Water which crystallizes on cooling but on heating “pre-melts” around −18 °C with the enthalpy *∆H*_prem._; in Fig. 7, it is marked by right-slanting lines.
- Water which does not crystallize on cooling and its fraction in a system is described with the formula:

6 $$ {w}_{\mathrm{b}.\mathrm{w}.}={w}_{\mathrm{H}2\mathrm{O}}\cdot \left(1-\frac{\varDelta {H}_{\mathrm{prem}.}+\varDelta {H}_0}{\varDelta {H}_{\mathrm{H}2\mathrm{O}}}\right) $$

where *w*_b.w._ is a weight fraction of water bound to a polymer, *w*_H2O_ is a total weight fraction of water in a hydrogel, and the enthalpy of melting of ice for distilled water *∆H*_H2O_ equals 333 J/g.

**Fig. 8:**
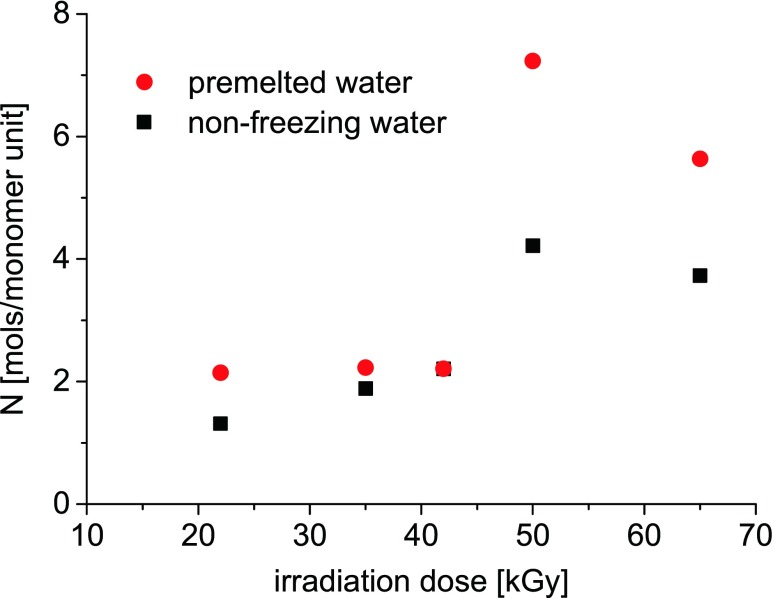
Amount of water molecules hydrating monomer unit of PVME for particular hydrogel samples estimated as amounts of non-freezing water molecules (*squares*), the amount of pre-melted water molecules (*circles*)

The fractions of the non-freezing bound water *w*_b.w_ in the hydrogel samples were calculated with the use of the formula (6). Then, *w*_b.w_ substituted *w*_H2O_^liq^ in the formula (3) to be expressed as a hydration number *N* (shown in Fig. 8).

Quantity of this part of water which exhibit pre-melting was also determined from the standard DSC thermograms and is plotted in Fig. 8 as an average number of water molecules per monomer unit of polymer network. One can notice that both values of *N* have a weak dependence on the irradiation dose for doses up to 42.5 kGy (for samples PVME-22, PVME-35, and PVME-42, *N* is around 2) and for hydrogels crosslinked with higher irradiation doses these values are higher; in PVME-50, there are around four non-freezing water molecules and around seven pre-melting water molecules per monomer unit. In sample PVME-65, there are four non-freezing water molecules and around six pre-melting water molecules per monomer unit.

## Discussion

The general temperature dependence of the frequency of the mode ν_s_(CH_3_) is similar in all investigated systems. The onset of the blue shift of this band with temperature is located in the range −60 to −30 °C. Such evolution of the position of the ν_s_(CH_3_) band indicates that on heating from −60 to 0 °C, hydration of PVME segments gradually increases. It is important to note, that the blue shift of this band is accompanied by simultaneous decay of the intensity of the collective band of ice (Fig. 2). The coincidence of these two phenomena points out that when ice starts to pre-melt the produced liquid water immediately constitutes hydrogen bonds with hydrophilic sites at polymer chains.

It is also worthy to note that the increasing hydration of the polymer segments coincides with increasing segmental mobility of PVME. This can be explained by the fact that the *α* process is associated with the pre-melting of ice, as it was shown in our previous work [9]. Hence, considering calorimetric, dielectric and Raman results the most probably scenario goes as follow: heating of the hydrogel over −60 °C activates the polymer chains mobility which is, however, initially restricted by presence of the ice crystals. Upon further heating, the mobile polymer segments move also some of the neighboring molecules of frozen water what triggers the process of the pre-melting. In the next step, the molecules of liquidized water establish hydrogen bonds with polymer chains. From that point, the polymer chains can move cooperatively with H-bonded water molecules. That cooperativity was manifested as an increase in dielectric strength of the *α* process of PVME, what we have reported in our previous work [9].

**Fig. 9:**
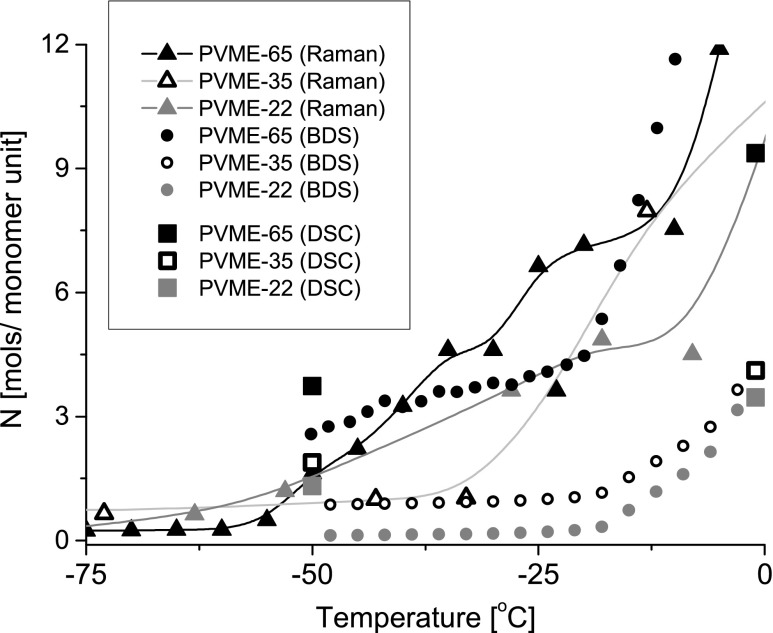
The summary plot presenting the evolution of the hydration number *N* of the PVME monomer unit in PVME-22 (*grey*), PVME-35 (*empty*), and PVME-65 (*black*) with temperature, determined by DSC (*squares*), BDS (*circles*), and Raman spectroscopy (*triangles*); the values obtained by DSC are plotted at −50 °C (non-freezing bound water) and at −1 °C (non-freezing bound water + pre-melted water). The temperatures were selected arbitrary, −50 °C as the lowest point measured by DSC and −1 °C as the highest point below the fusion of ice; the *lines* are plotted for eye guidance of the points obtained from Raman spectroscopy

We have studied with use of Raman spectroscopy, dielectric spectroscopy, and calorimetrically, how the amount of non-crystallized water in PVME hydrogels increases with temperature between −50 and 0 °C. The outcomes of our analyses are summarized in Fig. 9, which presents the hydration number *N* determined with these methods in three hydrogels: PVME-22, PVME-35, and PVME-65. Values obtained by DSC are shown only at −50 °C (the fraction of unfrozen bound water) and at −1 °C (the fraction of unfrozen bound water plus pre-melted water). These temperatures were selected because −50 °C was the lowest point measured by DSC and −1 °C was the highest point below the fusion of ice.

The comparison reveals good qualitative agreement between the results obtained with different techniques. In all studied samples we observe the presence of some fraction of liquid water at −50 °C, i.e., there are around 1–2 water molecules per monomer unit in PVME-22 and PVME-35 and three to four water molecules per monomer unit in PVME-65. Above −50 °C, the hydration number gradually increases with temperature in all hydrogels due to the pre-melting of ice, but this increase is the most pronounced in PVME-65. The differences between *N* in particular hydrogels should be related to different density of their polymer networks. Hence, the higher amount of unfrozen bound water in the denser crosslinked samples can be a result of confinement of water molecules in tight meshes of these networks*.* It may imply that in a very dense network, in which some quantity of water is trapped in nanoscopic voids, the nanodroplets of water may be formed. Such nanodroplets can reveal thermodynamic properties very different to these of the “normal” ice and as a consequence melt much below 0 °C [18]. It should be noted that the concept of hydration number *N* is applicable principally to homogenous liquid systems. As we already mentioned the process of radiational crosslinking composes of generation and recombination of macroradicals. These processes lead to formation of defects such as additional end groups and nods of a polymer network. As a consequence, the thus obtained hydrogel constitutes a highly inhomogeneous system. The hydration of both types of defects is different from the hydration of a monomer unit. As a result, the determined by us hydration numbers are in fact the averaged hydration numbers of various species existing in the PVME networks (regular monomer unit, nod of a network, free end group). These effects are obviously the most apparent in the sample PVME-65.

The applied techniques are tools able to observe different properties of frozen and unfrozen water: their vibrations, rotations in electric field, and thermal properties. From Fig. 9 it is clear that evolution of unfrozen water with temperature seen by all these techniques is qualitatively very similar. Quantitatively, there is also a good agreement between hydration number *N* determined calorimetrically and *N* derived from dielectric depolarization. Yet, one may notice some inconsistency concerning the Raman results with those obtained by DSC and BDS. Differences in quantitative data can be due to different accuracy of each technique and assumptions used for the analysis. Raman spectroscopy is especially sensitive to local conformation of molecules (here, the environment of methoxy groups). Increase in a number of unfrozen water molecules is therefore “early detected” and the accuracy of the determination of *N* is the best in the low temperature range (where the fraction of liquid is the smallest). On further heating, the additional molecules of liquid water are more distant from the polymer chains and thus they are changing the vibrational states of CH_3_ groups in smaller and smaller degree. On the contrary, the dielectric spectroscopy, in which rotations of dipoles are observed, is the most uncertain in the low temperature region. As we already concluded, the water molecules which pre-melt quickly arrange hydrogen bonds with hydrophilic sites at polymer chain and therefore their rotations are strongly restricted [6]. Hence, they do not contribute to dielectric depolarization, and as a result, those water molecules are “invisible” in dielectric spectra.

## Summary and conclusions

We studied states of water at temperatures below 0 °C in PVME hydrogels of various crosslinking degrees. With the use of dielectric spectroscopy, we estimated fraction of water able to rotate in the range of negative temperatures, while the DSC allowed us to determine the amounts of both non-freezing and pre-melting water. We found a good agreement between the DSC and the BDS-based estimations of the hydration number of the PVME monomer unit.

Thanks to high sensitivity of Raman spectroscopy to a local environment of molecules, we perceived that between −163 and −60 °C, the PVME chains are fully dehydrated. We noticed that the pre-melting process starts in significantly lower temperature than was observed by BDS and DSC, i.e., around −60 °C and proceeds with simultaneous reconstruction of hydrogen bonding between the polymer and water molecules.

Based on presented herein results, we postulate the triggering mechanism of the pre-melting process. Namely, we suggest that above ca. −60 °C, the first segmental motions of the polymer chain are activated. Next, these mobile segments move some of the neighboring molecules of frozen water, which triggers the process of the pre-melting.
